# The Sequence Effect Worsens Over Time in Parkinson’s Disease and Responds to Open and Closed-Loop Subthalamic Nucleus Deep Brain Stimulation

**DOI:** 10.3233/JPD-223368

**Published:** 2023-06-13

**Authors:** Yasmine M. Kehnemouyi, Matthew N. Petrucci, Kevin B. Wilkins, Jillian A. Melbourne, Helen M. Bronte-Stewart

**Affiliations:** a Stanford University School of Medicine, Department of Neurology and Neurological Sciences, Stanford, CA, USA; b Stanford University School of Engineering, Department of Bioengineering, Stanford, CA, USA; c Stanford University School of Medicine, Department of Neurosurgery, Stanford, CA, USA

## Abstract

**Background:**

The sequence effect is the progressive deterioration in speech, limb movement, and gait that leads to an inability to communicate, manipulate objects, or walk without freezing of gait. Many studies have demonstrated a lack of improvement of the sequence effect from dopaminergic medication, however few studies have studied the metric over time or investigated the effect of open-loop deep brain stimulation in people with Parkinson’s disease (PD).

**Objective:**

To investigate whether the sequence effect worsens over time and/or is improved on clinical (open-loop) deep brain stimulation (DBS).

**Methods:**

Twenty-one people with PD with bilateral subthalamic nucleus (STN) DBS performed thirty seconds of instrumented repetitive wrist flexion extension and the MDS-UPDRS III off therapy, prior to activation of DBS and every six months for up to three years. A sub-cohort of ten people performed the task during randomized presentations of different intensities of STN DBS.

**Results:**

The sequence effect was highly correlated with the overall MDS-UPDRS III score and the bradykinesia sub-score and worsened over three years. Increasing intensities of STN open-loop DBS improved the sequence effect and one subject demonstrated improvement on both open-loop and closed-loop DBS.

**Conclusion:**

Sequence effect in limb bradykinesia worsened over time off therapy due to disease progression but improved on open-loop DBS. These results demonstrate that DBS is a useful treatment of the debilitating effects of the sequence effect in limb bradykinesia and upon further investigation closed-loop DBS may offer added improvement.

## INTRODUCTION

The sequence effect is the progressive deterioration in ongoing movement that is not related to peripheral muscle fatigue [1] and it appears to be specific to Parkinson’s disease (PD) [2, 3]. Although initially thought to be a feature of more advanced stage PD, the sequence effect in speech and limb movement has been documented in very early stages of PD in drug naïve people [4–7]. The sequence effect is one of the most debilitating features of PD and a significant source of morbidity: the progressive shortening of step length may be associated with freezing of gait (FOG), which frequently results in falls [8–12]. It has been observed that FOG frequently results from the superimposition of the sequence effect on gait hypokinesia and that FOG likely will not occur in its absence [9–11]. The sequence effect in limb bradykinesia is evident in both the amplitude and frequency of ongoing movements and impairs the ability to write, use a computer keyboard and manipulate objects such as tools or buttons [3, 13–18]. The sequence effect in speech in PD causes reduced intelligibility, articulatory imprecision, and altered rates of speech, which affects up to 90% of people with PD, worsens with disease progression, and causes significant morbidity [19, 20].

Although different components of bradykinesia can be treated with levodopa and/or deep brain stimulation (DBS), a critical unmet need for improving the lives of people with PD is that the sequence effect does not respond to dopaminergic medication [1, 3, 4, 20–22]. No study to our knowledge has directly examined the effect of DBS on the sequence effect in PD. Few studies have specifically examined whether the sequence effect improves during DBS [18, 23].

Our goal in this study was to investigate whether the sequence effect improved during subthalamic nucleus (STN) DBS in people with PD and whether there was a ‘dose” or DBS intensity dependence. We measured the sequence effect during repetitive wrist flexion-extension, correlated it with the Movement Disorders Society-Unified Parkinson’s Disease Rating Scale (MDS-UPDRS III) and with its sub-score of lateralized bradykinesia and demonstrated that it became worse over time in the off therapy state, but improved with STN DBS.

## MATERIALS AND METHODS

### Participants

Twenty-one individuals (5 female) with clinically established PD underwent bilateral implantation of DBS leads (model 3389, Medtronic PLC) in the STN. The two leads were connected to the implanted investigative neurostimulator (Activa^®^ PC+S, Medtronic PLC, FDA Investigational Device Exemption (IDE) approved). The preoperative selection criteria included: 1) participants with a diagnosis of idiopathic PD, 2) documented improvement in motor signs on versus off dopaminergic medication, 3) the presence of complications of medication such as wearing off signs, 4) fluctuating responses and/or dyskinesias, and/or medication refractory tremor, and/or impairment in the quality of life on or off medication due to these factors, 5) ability and willingness to return for study visits (initial programming and subsequent 3-month interval clinical DBS visits), and 6) age greater than 18 years old. Exclusion criteria for participants was as follows: dementia, untreated psychiatric disease, Hoehn and Yahr stage 5 on medication, age greater than 80 years old, major surgical morbidities, such as severe hypertension, coagulopathy, and certain metabolic conditions that might increase the risk of hemorrhage or other surgical complications, presence of a cardiac pacemaker/defibrillator, and inability to understand/sign consent forms. The surgical technique has been previously described [26, 27]. Upon termination of the open-loop study, one participant in the cohort (male) underwent an IPG replacement from the Activa PC+S to another investigative neurostimulator (Summit™ RC+S), which is compatible with the same leads, and allowed him to complete the closed-loop study. All participants gave written consent to participate in the study, which was approved by the Food and Drug Administration (FDA) and the Stanford University School of Medicine Institutional Review Board (IRB). The clinical trial numbers for this study are NCT01990313 and NCT02384421.

### Experimental protocol

#### Off stimulation longitudinal testing

Experiments were conducted at initial programming (IP, before the initial activation of the DBS system), which took place 1 month after implantation of the DBS leads, and subsequently at 6 months intervals out to 3 years after the IP visit; the maximum number of total visits was 7 (IP, 6, 12, 18, 24, 30, 36 months). All experimental testing was done in the off-medication state, which entailed the withdrawal of long-acting dopamine agonists for 48 hours, dopamine agonists and controlled release carbidopa/levodopa for 24 h, and short acting medication for 12 h prior to the study visit. At the follow-up visits, stimulation was turned off for 60–75 min. The participant then performed a single trial of repetitive wrist-flexion extension (rWFE) task, which we have previously validated as a measure of bradykinesia in PD [6, 28–30]. Participants were instructed to remain seated and as still as possible with their eyes open, and after a “Go” command, to flex and extend the hand at the wrist joint as quickly as possible and to stop only when instructed; the forearm was flexed so that the elbow was angled at 90°. The movement was self-paced and lasted 30 s. MDS-UPDRS III was performed by a certified rater pre-operatively (on and off medication), off medication at IP, and then off all therapy at all follow-up visits after DBS had been turned off for at least 60 min. The wrist assessed first was randomized regarding the more or less affected side.

#### Titrations

A sub-cohort of 10 individuals (3 female) completed a stimulation amplitude titration experiment, off medication. These participants had been implanted with investigative deep brain neurostimulators that recorded STN local field potentials synchronously with kinematic signals during a variety of tasks. Testing was done once optimized on DBS settings and 1.5–3 years after their initial programming visit, and at a time when they tolerated changing of stimulation. Participants in this study did not have tremor that superseded the entire trial of the WFE task. In some subjects, only one wrist was assessed if the subject had excess neural artifact that resulted in that limb/STN being excluded from the experiment. Furthermore, due to protocol time restrictions and fatigue for the subjects, one or both wrists were assessed (randomized in order). Participants performed five trials of the rWFE task, where a single trial was performed unilaterally on one randomized side. Each trial was performed during randomized presentations of STN DBS at 0% (no DBS), 25%, 50%, 75%, and 100% of V_max_. V_max_ represented the clinically equivalent DBS intensity using a single active electrode, with which neurostimulation improved bradykinesia to a similar degree to that observed when using the clinical DBS intensity delivered through one or multiple electrodes. Each participant performed one round of the entire experiment at the designated visit.

#### Closed-loop DBS

In one participant, data were collected during the rWFE task in three stimulation conditions from the same wrist: off stimulation (one trial), clinical open-loop stimulation (olDBS) (one trial), and neural closed-loop stimulation (NclDBS) (five trials, each with a varying stimulation delay period). The NclDBS condition used the beta burst driven adaptive closed-loop algorithm [32] with the addition of this stimulation delay period control parameter (i.e., the minimum duration of time paused after one stimulation decision before the next allowable stimulation decision). The stimulation delay period was varied from 800–2000 ms between stimulation decisions. The inter-trial resting time between trials was about 5 min. The stimulation parameters for olDBS were (LSTN: C+,1-,2- at 4.8 mA; RSTN: C+, 8-,9-at 5.2 mA; 140 Hz) and NclDBS (LSTN: C+, 1-, 3.2–4.0 mA; RSTN: C+, 9-, 3.4–4.2 mA; 140 Hz).

### Data acquisition and analysis

#### Kinematic data acquisition

Movement was measured using solid-state gyroscopic wearable sensors (sampled at 1 kHz) attached to the dorsum of each hand (Motus Bioengineering, Inc, Benicia, CA) and with synchronized video recordings from a USB web camera (C930e, Logitech, Lausanne, Switzerland). Sampling rates for the gyroscope and video data were 1 kHz and 30 frames per second, respectively.

#### Kinematic data analysis

For each movement epoch, an automated algorithm was used to quantify the sequence effect. The angular velocity data was low-pass filtered in MATLAB using zero-lag 4th order Butterworth filters with a 4 Hz cutoff frequency at the above sampling frequency. Peaks for analysis were chosen based on the maximum angular velocity between each zero crossing for each cycle of flexion-extension. For traces with excess tremor that required extra smoothing to find accurate zero crossings, the angular velocity data was low-pass filtered again using zero-lag 4th order Butterworth filters starting with a 2.5 Hz cutoff frequency, and if subsequent filtering was required the data was re-filtered using a cutoff frequency that decreased by 0.5 Hz until no further filtering was required or the cutoff frequency decreased to 0 Hz, at which case the trace was not usable as zero crossings could not be accurately detected. This filtered trace was only used to identify the zero crossings, which were then applied to the peak detection on the original angular velocity data.

Since some trials displayed multiple epochs of sequence effect in which the participant was able to reset following initial decrements in angular velocity, an automated algorithm was used to determine if the trial should be broken up into one or multiple epochs. First, a 3-point moving average was calculated on the angular velocity peaks to better dynamically visualize and model the trend in behavior as well as filter out periodic fluctuations and noise. To further protect the sequence effect models against overfitting from sudden behavioral fluctuations, the percent change was calculated on the moving average of peaks in two ways: between the current peak and the subsequent peak (denoted as PC1) as well as between the current peak and the next 2 peaks (denoted as PC2). A negative percent change represented a smaller angular velocity from previous, typically seen during the sequence effect epoch, and a positive percent change represented a larger angular velocity from previous, which if large enough represented the end of a sequence effect epoch. Upon inspection across the cohort’s rWFE traces, a threshold of 20% was empirically derived, where once the percent change in angular velocity crossed this threshold, a sequence effect epoch had been completed.

Following epoching, an exponential curve was fit to the first epoch of decay in a trace using the following criteria: for those where the maximum angular velocity is greater than 100 degrees/s and there are at least 10 full cycles of rWFE present, the initial point of the fitted exponential was chosen as the maximum of the first 10 peaks. For traces where the maximum angular velocity was less than 100 degrees/s and there were at least 5 full cycles of rWFE present, the initial point was chosen as the maximum of the first 5 peaks. For traces that fit neither criterion, the initial point was chosen as the first peak. The algorithm starts a new epoch when either the PC1 or PC2 trace crossed the 20% threshold and the current peak is at least 40% of the first epoch’s maximum peak (representing an accurate pick up in behavior). When this is true, the first epoch will end with the last point with negative percent change prior to crossing the 20% threshold and the second epoch will begin to be fit with an exponential function, and this process repeats until the entire trace’s sequence effect epochs have been fit with exponential curves.

The sequence effect behavior in each epoch was modeled using the exponential decay function equation:

(1) $$y={\mathrm{Ce}}^{-\mathrm{kx}}$$

Where *y* = movement amplitude, *C* = the model intercept, *x* = time, and *k* = the slope of the decay.

The slope of the decay was normalized by the initial angular velocity, *A*, to take into account the speed of the movement. A natural log was then used to normalize the distribution of the data:

(2) $$\ln\left(\frac{A}{\left|k\right|}\right)$$

Finally, to express the sequence effect metric as a percentage where a higher number was indicative of greater (i.e., worse) sequence effect, the inverse of Equation 2 was used and multiplied by 100:

(3) $$100\left(\frac{1}{\ln\left(\frac{A}{\left|k\right|}\right)}\right)$$

For cases where the epoch is overall increasing and an exponential growth curve was more suitable for modeling the behavior, the epoch was modeled using Equation 1 with a positive exponent, and the sequence effect metric was still calculated with Equation 3, but in this case the *A* coefficient in the metric was taken as the maximum angular velocity that the participant obtained in the epoch and *k* represented the slope of growth. Periods during the epoch in which tremor superseded the movement were also removed from analysis. Though this proposed modeling algorithm ran automatically, for cases where the outputted sequence effect model was visually not representative of the data, manual adjustments were made, such as shifting the starting point or increasing the percentage threshold. Finally, though in some traces there were multiple epochs of sequence effect fit by the algorithm, to avoid confounding the sequence effect metric only the first decay epoch was denoted as the primary epoch of sequence effect for analysis. The sequence effect was correlated with the MDS-UPDRS III lateralized bradykinesia subscore, and the items included 3.4 (finger tapping), 3.5 (hand movements), 3.6 (pronation-supination), 3.7 (toe tapping), and 3.8 (leg agility) for each side.

### Statistical analysis

Statistics were computed using MATLAB (version 9.9, The MathWorks Inc. Natick, MA, USA). Pearson correlations were used to assess associations between the sequence effect during rWFE and both total MDS-UPDRS III scores and the bradykinesia sub-score. A linear mixed effects regression model was performed to analyze the effect of time (in months) on sequence effect. In this case, the sequence effect metric was included as the dependent variable and visit month was used as a fixed effect with subject as a random intercept and time as a random slope. For analysis of the titrations results, Kolmogorov-Smirnov tests and theoretical-sample quantile plots were used to assess the normality of the distribution of sequence effect at each stimulation condition. Based on the titrations experiment results, a repeated measures ANOVA was used to compare the effect of stimulation level on sequence effect across all STNs at the five stimulation conditions. Paired *t*-tests were used *post hoc* to compare the difference in sequence effect between the 0–25%, 0–50%, 0–75%, and 0–100% V_max_ DBS conditions. A raw *p* value <0.0125 was used for significance after Bonferroni correction for multiple comparisons (N = 4).

## RESULTS

Kinematic data from 42 hands of 21 well-characterized individuals with Parkinson’s disease, off therapy, were included in the analysis. Table 1 details the demographic data of the participants. For the longitudinal study, the average number of visits was 5.2±1.6 over a span of up to 36 months after IP, and the average duration after IP of the final visit was 27.7±10.1 months (Table 1). For the titrations experiment, kinematic data from 16 hands of 10 well-characterized individuals with PD were included in the analysis. This experiment was conducted 2.5±0.6 years after the participant’s initial programming visit. The mean age of this sub-cohort was 55.3±9.0 years, and mean disease duration was 8.9±3.1 years. Their percent improvement from OFF to ON DBS was 72.4% ±14.4%, on the day of the experiment. Participants were off medication.

**Table 1:** Participant demographics

Participant	Age	Sex	Disease duration at IP	Year of PreOp	PreOp % Improvement in (MDS)-UPDRS III from medication*	IP MDS-UPDRS III	# of Visits	Month (after IP) of Last Visit	Years (after IP) of Titration Experiment
1	72.9	M	10	2012	24.1	60	4	24	–
2	52.8	M	5	2013	66.7	20	7	36	3.3
3	62.5	M	3	2013	34.6	36	6	36	3.3^$^
4	65.7	M	5	2013	44.8	38	4	36	3
5	58.1	M	3	2013	41.0	43	7	36	2.8
6	42.2	M	4	2013	79.3	51	5	30	2.8^$^
7	69.0	M	10	2013	31.7	43	2	6	–
8	53.5	M	7	2013	44.2	61	6	36	2.6^$^
9	72.2	M	9	2013	51.1	45	6	30	–
10	58.6	F	10	2013	81.8	42	6	36	–
11	54.5	M	11	2014	64.3	60	2	6	–
12	34.3	M	7	2014	62.7	70	6	30	1.9
13	57.3	M	6	2014	62.9	56	3	12	–
14	67.6	F	6	2014	43.2	12	7	36	–
15	50.6	F	9	2014	48.0	51	6	30	1.9^$^
16	61.9	M	14	2014	42.9	44	4	18	–
17	52.0	F	12	2015	82.4	22	7	36	1.8
18	66.1	M	7	2015	80.4	33	5	30	–
19	56.8	F	11	2015	52.6	34	7	36	1.6
20	55.0	M	9	2017	83.3	22	4	18	–
21	50.1	M	10	2018	18.4	48	5	24	–
Average±SD	57.8±9.6	8.0±3.3	54.3±19.8	42.4±15.1	5.2±9.3	27.7±10.1	2.5±0.6

IP, initial programming; UPDRS, Unified Parkinson’s Disease Rating Scale; MDS, Movement Disorder Society. ^*^Participants 1–5 were assessed pre-operatively with the UPDRS III, and 6–21 with the MDS-UPDRS III. ^$^Indicates only 1 hand tested.

### Quantification of sequence effect epochs

**Fig. 1:**
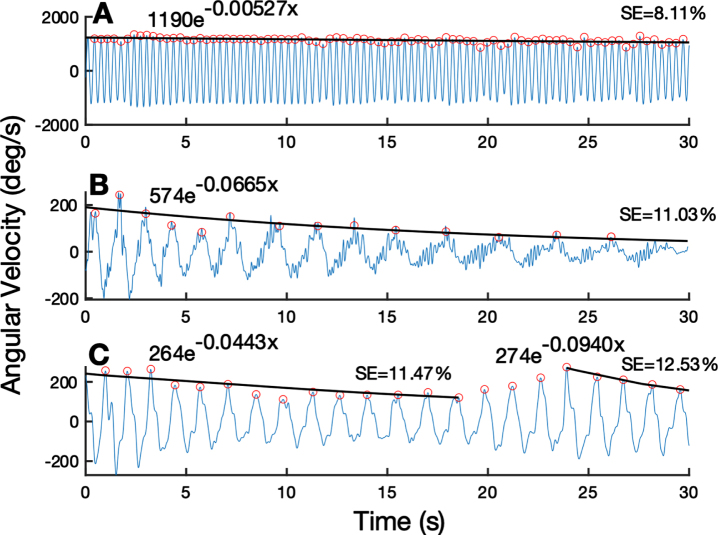
Quantification of sequence effect on example WFE traces. (A) WFE trace with minimal sequence effect. (B) WFE with one epoch of substantial sequence effect for the entire trace. (C) WFE split into two epochs based on reemergence of performance around 25 seconds. Fitted exponential line is shown in black. Detected peaks shown by orange open circles. Exponential fit function and subsequent sequence effect (SE) metric shown above each trace.

Figure 1 presents the output of the algorithm, which fits the rWFE angular velocity trace with a characteristic exponential decay curve. Figure 1A displays the behavior of an individual with minimal sequence effect, represented by a lower sequence effect metric. Figure 1B shows an individual with a larger sequence effect whose dynamics were modeled by one exponential decay curve. Figure 1C portrays an example of an individual who experienced two epochs of sequence effect as modeled using two separate exponential decay curves by the algorithm. At the initial visit of the longitudinal off-stimulation study, 25 hands showed only one epoch of sequence effect and 17 exhibited multiple epochs.

Across the 21 participants throughout the 3 years of repeat visits, there were 212 total trials (Table 2). 134 trials showed only one epoch of sequence effect and 78 trials showed multiple epochs of sequence effect.

**Table 2:** Trials over time

Month	Data Files
1	37
6	41
12	38
18	29
24	28
30	21
36	18

### Sequence effect is related to overall motor impairment and bradykinesia

**Fig. 2:**
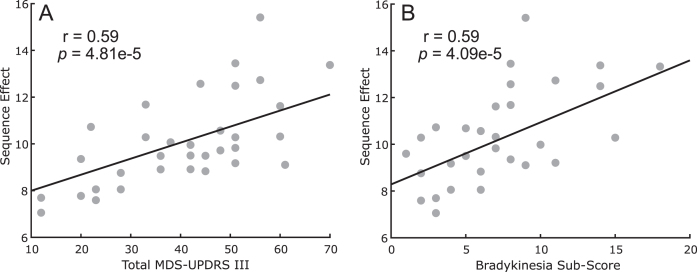
Scatter plot between sequence effect during rWFE and (A) total MDS-UPDRS III scores and (B) lateralized bradykinesia sub-scores.

Figure 2 shows the comparison between the measured sequence effect (*n* = 42 hands) versus the total MDS-UPDRS III score (Fig. 2A) and versus the lateralized bradykinesia sub-score for the tested side (Fig. 2B) at the initial programming visit. A higher (i.e., worse) sequence effect was associated both with greater total motor impairment (*r* = 0.59, *p* = 4.81e-5) and with upper and lower extremity lateralized bradykinesia (*r* = 0.59, *p* = 4.09e-5).

This relationship was confirmed at each 6-month timepoint for both total MDS-UPDRS III score: (6m: *r* = 0.55, *p* = 2.80e-4, 12m: *r* = 0.52, *p* = 8.15e-4, 18m: *r* = 0.41, *p* = 0.049, 24m: *r* = 0.45, *p* = 0.015, 30m: *r* = 0.62, *p* = 0.0048, 36m: *r* = 0.69, *p* = 0.0031) and lateralized bradykinesia sub-score for the tested side (6m: *r* = 0.68, *p* = 1.47e-6, 12m: *r* = 0.59, *p* = 9.63e-5, 18m: *r* = 0.61, *p* = 8.61e-4, 24m: *r* = 0.49, *p* = 0.0067, 30m: *r* = 0.79, *p* = 6.19e-5, 36m: *r* = 0.76, *p* = 2.24e-4).

### Sequence effect worsens over time

**Fig. 3:**
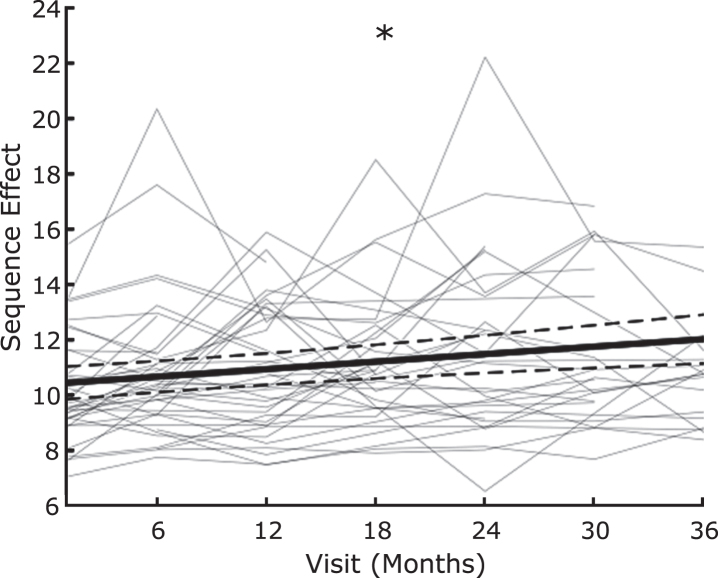
Sequence effect worsens over time off therapy. Average slope of change over time (thick black line) with individual data overlaid as line plots (light gray) of sequence effect. * indicates significant change over time. Dashed lines represent the 95% confidence interval of the slope estimate.

Figure 3 shows the sequence effect across the longitudinal group after performing the rWFE task off therapy, at up to 7 timepoints out to 3 years after the initial programming visit.

The sequence effect significantly increased over time, off therapy (β= 0.0453 (95% CI: 0.0328 to 0.0578), *t* = 3.62, *p* = 0.00037).

### STN DBS improves the sequence effect

**Fig. 4:**
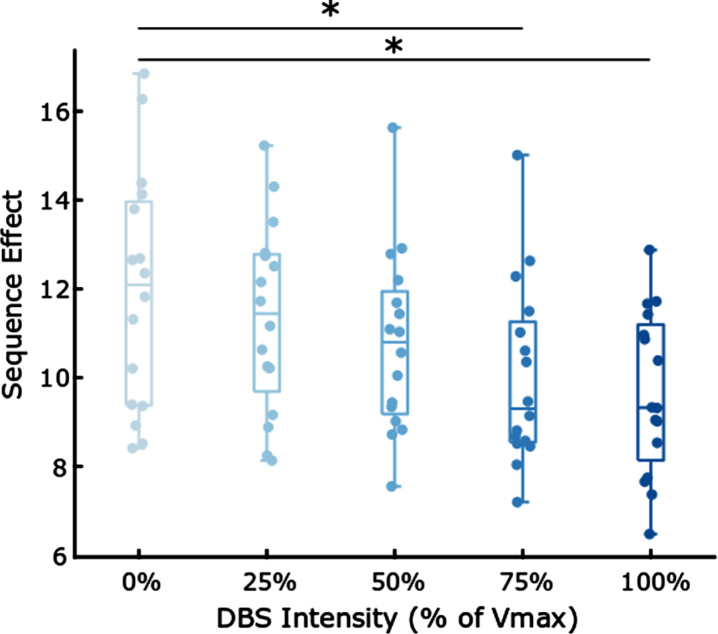
Boxplots comparing the sequence effect at different intensities of DBS. * indicates *p* < 0.05, Bonferroni corrected.

Figure 4 shows the box plot of sequence effect (*n* = 16 hands) during randomized presentations of STN DBS at 0%, 25%, 50%, 75%, and 100% of the maximum intensity (V_max_).

Table 3 demonstrates that increased intensities of DBS were associated with a decrease (i.e., improvement) in sequence effect during rWFE [F(4,60) = 3.01; *p* = 0.0233]. *Post hoc* paired *t*-tests tests showed significant decreases in the sequence effect during the 75% and 100% V_max_ stimulation conditions: 0% and 75% V_max_ (t(15) = 3.14, p = 0.0068), 0% and 100% V_max_ (t(15) = 3.80, p = 0.0017). Therefore, olDBS reduced the sequence effect in a dose-dependent manner, with significant differences occurring at 75% and 100% V_max_.

**Table 3:** Dose-dependent reduction in mean sequence effect

Stimulation Percentage	Mean Sequence Effect
0	11.85
25	11.35
50	10.77
75	10.00
100	9.65

### Sequence effect improved during neural closed-loop DBS in one participant

**Fig. 5:**
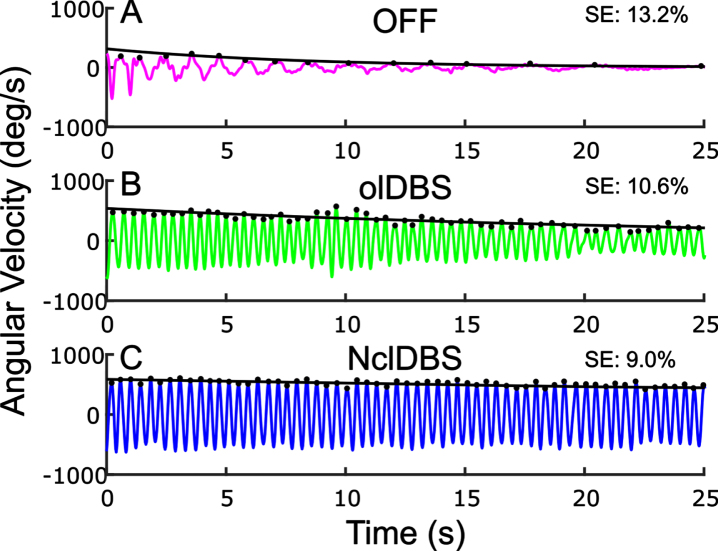
Example of Wrist Flexion Extension (WFE) (A) OFF therapy, (B) open-loop clinical stimulation, (C). neural closed-loop stimulation. Peaks from each WFE cycle indicated by black dots with the fitted exponential curve overlaid.

Figure 5 demonstrates the effect of open and closed loop STN DBS on the sequence effect during rWFE in one participant. The sequence effect improved ON clinical olDBS compared to OFF therapy (Fig. 5A, B) and improved further ON NclDBS (Fig. 5C).

NclDBS improved the sequence effect in 8 out of the 10 NclDBS conditions across various delay periods between the two hands (Table 4).

**Table 4:** Neural closed-loop parameters

Right Hand			Left Hand		
Condition	Delay (ms)	Sequence Effect	Condition	Delay (ms)	Sequence Effect
OFF	–	13.23%	OFF	–	13.55%
olDBS	–	10.55%	olDBS	–	10.03%
NclDBS	800	9.52%	NclDBS	800	9.70%
NclDBS	1000	9.74%	NclDBS	1000	9.84%
NclDBS	1200	9.46%	NclDBS	1200	10.51%
NclDBS	1500	9.03%	NclDBS	1500	10.59%
NclDBS	2000	9.82%	NclDBS	2000	9.46%

## DISCUSSION

This study found that an objective normalized metric of the sequence effect in upper extremity bradykinesia strongly correlated with the total MDS-UPDRS III score and with the upper and lower extremity lateralized MDS-UPDRS III bradykinesia subscore in people with PD. The sequence effect worsened over time in a longitudinal cohort studied off therapy up to three years after initial programming, demonstrating that it reflects the progression of PD. The sequence effect in limb bradykinesia improved during STN continuous open loop DBS in a dose-dependent manner and in one participant the sequence effect improved further on neural closed loop DBS.

### Measuring the sequence effect in limb movement

One of the difficulties in assessing evidence of the sequence effect and its response to therapies in PD is the paucity of measurement tools that can differentiate the sequence effect from other metrics such as amplitude and frequency. The MDS-UPDRS motor subscale (MDS-UPDRS III) groups together assessments of impairment in amplitude, frequency, and sequence effect into one integer for each item related to limb bradykinesia. Consequently, one cannot discern a specific metric of the sequence effect from the MDS-UPDRS III. Even with quantitative measures, the measure of the sequence effect has varied from a comparison of the first and last multiple of cycles of repetitive movement or gait [32] to linear regressions of time series data [7, 12, 33]. We determined that an exponential fit best represented the sequence effect in time series data for instrumented repetitive wrist flexion-extension. The exponential curve best representing the sequence effect was personalized to each participant’s wrist flexion extension data by an automated algorithm. The automated algorithm fit an exponential decay or growth function to epochs of the angular velocity trace and the majority of trials (63%) demonstrated only one epoch. For all cases we designated the first epoch as the outcome variable for the sequence effect.

### The sequence effect is a validated measure of PD motor disability

In this study the sequence effect was significantly correlated with overall PD motor disability (MDS-UPDRS III), and this was not solely due to lower angular velocities in later stages of disease, as it was normalized by initial peak velocity. The sequence effect was also significantly correlated with the lateralized bradykinesia sub-score on the MDS-UPDRS III; this is expected as bradykinesia is defined as “slowness of movement and decrement in amplitude or speed (sequence effect) as movements are continued”, which is similar to its definition in the first formal diagnostic criteria for PD [13, 35]. In this study the sequence effect explained about 35% of the overall MDS-UPDRS III upper and lower extremity lateralized bradykinesia integer sub-score, which reflects other components such as the amplitude and speed of the movement. This quantitative, normalized metric of the sequence effect is a validated measure of total motor disability and of bradykinesia and may be a useful metric for outcomes of clinical trials.

Previous literature suggests that the sequence effect appears to be specific to PD and multiple system atrophy but does not appear to be a major feature of progressive supranuclear palsy (PSP), Huntington’s disease, or dystonia [1–3, 33]. The sequence effect in finger tapping movements has been suggested as a tool to differentiate PSP from PD [3]. It has not been found to correlate with peripheral fatigue or mood [1, 22]. The sequence effect in limb bradykinesia has been shown to be a feature of early-stage PD in sequential arm movements, in the Purdue-Pegboard Test, and in finger tapping [4, 6, 7, 33]. In this study, the sequence effect was evident across a broad spectrum of disease severity, even in participants of later stages of PD who present more severe bradykinesia (total MDS-UPDRS III scores >50).

### The sequence effect improved on open and closed-loop STN DBS

One of the debilitating features of the sequence effect in PD is that it does not improve on levodopa or during repetitive transmagnetic stimulation (rTMS) [1, 3, 20–22, 35, 36]. Few studies have measured the sequence effect during DBS, although it is well established that DBS improves the overall assessment of bradykinesia from the MDS-UPDRS III. In this study we used randomized presentations of different intensities of STN continuous olDBS, using a single monopole, and found a dose-dependent improvement in the sequence effect in limb bradykinesia during STN olDBS, which was significant at 75% and 100% of the matched clinical DBS intensity, when using monopolar stimulation. In one subject NclDBS further improved the sequence effect compared to clinical olDBS in 8 out of the 10 NclDBS conditions. These results are similar to a case report we previously published, which demonstrated the superiority of an hour of fully embedded NaDBS (Activa^TM^ PC+S-NexusE, Medtronic PLC) for the sequence effect in limb bradykinesia compared to olDBS [24]. We have also demonstrated that NaDBS was superior to olDBS for the sequence effect in a stepping in place task in a person with PD, gait impairment and FOG [25]. Future work can look into evaluating whether the improvement in the sequence effect from NclDBS is more evident over long-term NclDBS and whether this will manifest as a clinically meaningful improvement to people with PD.

### Neural basis of the sequence effect and potential mechanism for the therapeutic effect of DBS

Several studies suggest that the sequence effect may arise from pathological neural activity in cortical, subcortical and cerebellar circuitry that contribute to central drive, motor sequencing, sensorimotor integration, timing cues, and updating of the motor set [1, 5, 7, 22, 37–41]. Deficits in sensorimotor integration in PD are proposed to be related to irregular and bursting neuronal firing in basal ganglia, pre-motor, and supplementary motor circuitry [42–44]. Irregular and bursting neuronal firing patterns increase on medication, which may further impair sensorimotor integration and may contribute to the lack of improvement in the sequence effect from medication [45, 46]. Recently, Lofredi et al demonstrated that the duration of prolonged low beta (13–20 Hz) bursts predicted the degree of the sequence effect in limb movement [33]. We demonstrated that STN DBS shortened resting state beta band burst durations in a dose-dependent manner [47] and that both 60 Hz and 140 Hz DBS shortened prolonged beta burst durations in people with PD and FOG, while improving their FOG [48]. We also demonstrated that the degree of improvement in angular velocity of the rWFE task at different intensities of DBS was related to the degree of shortening of beta band burst durations measured during the task [49]. This suggests that one mechanism for the therapeutic effect of DBS on the sequence effect may be in its ability to attenuate pathological beta burst durations. However, medication may also shorten beta burst durations and medication does not improve the sequence effect, so more investigation into other mechanisms are needed.

### Limitations

The experiments were all performed off medication and thus we cannot assert whether or not medication improved the sequence effect of this particular task, calculated by this metric. The study was focused on a novel metric of the sequence effect, its validation as a measure of bradykinesia and overall PD disease severity, and the effect of DBS. The correlations with bradykinesia and MDS-UPDRS III were performed before activation of DBS. To answer whether the sequence effect became worse over time and to examine the effect of DBS on the sequence effect, testing began after withdrawal of DBS for at least 60 min. After turning off STN DBS we have demonstrated that the resting state local field potential spectrum, and specifically beta band power, was stable and unchanged among recordings performed immediately (14 s) and every 15 min up to 60 min later, suggesting that this is enough time to wash out the effect of DBS on underlying pathophysiology [50]. Behavioral studies also suggest that the therapeutic effect of DBS on bradykinesia is largely washed out after 60 min off stimulation [51, 52]. Any residual effect of DBS on bradykinesia would have biased the data to show less progression compared to baseline and decreased effect of DBS, further supporting these findings.

An additional limitation is the presence of study dropout for the longitudinal OFF therapy protocol. Dropout was due to a combination of IPG replacement to a non-research device following battery depletion, moving out of state, or progression of disease that made OFF therapy testing intolerable. Due to their ability to account for missing data due to the participant dropout, linear mixed effect models were used in the analysis. Additionally, study exits prior to 3 years do not bias the results because participants are typically exiting the study once their bradykinesia has worsened so much that they are unable to perform the WFE task. These participants remaining in the study would have pushed the results even further in favor of the observed conclusion that sequence effect, which is related to bradykinesia, worsens over time.

One potential limitation for the titration experiment is there may be a ‘cumulative” effect of increasing doses of DBS on behavior as seen for attenuation of the beta band [29]; to avoid this, we presented varying intensities of DBS in a randomized manner, including an OFF DBS condition. The titrations experiment was conducted on a smaller subcohort of individuals that could tolerate a longer visit protocol, tolerated changing of stimulation well, and did not have artifact in their STN neural signal. Experiments were optimized for protocol time restrictions and avoiding participant fatigue, in which case participant wrist was randomized and either one or both wrists were assessed. As results were promising on this cohort, future experiments should be conducted on a larger cohort of individuals to investigate similar effects, and without the limitation of artifact-free STN LFPs, more participants could be included in the study.

Finally, as the neural closed loop sequence effect data is preliminary data taken from a single subject, it is difficult to conclude whether the noted behavioral improvement will be seen in a larger group of people with PD. For transparency, we included all data regardless of whether parameters such as stimulation delay are critical to the clinical significance.

### Conclusions

In this study we demonstrated that a quantitative metric of the sequence effect in upper limb movement strongly correlated with overall motor disability and upper and lower extremity bradykinesia in PD and worsened over time in a longitudinal cohort. In a careful examination of randomized presentations of different intensities of STN DBS, we showed for the first time that continuous open-loop DBS improved the sequence effect of limb bradykinesia in a dose-dependent manner. Finally, we presented evidence that closed-loop, or adaptive, DBS also improved the sequence effect and was slightly more efficacious than open-loop DBS in one participant. This suggests that STN DBS is a promising therapy to improve the sequence effect in bradykinesia for people with PD.

## FUNDING

This study was funded by the NIH Brain Initiative Grant 1UH3NS107709, NINDS Grant 5 R21 NS096398-02, PF-FBS-2024 from the Parkinson’s Foundation, Michael J. Fox Foundation (9605), Robert and Ruth Halperin Foundation, John A. Blume Foundation, and Helen M. Cahill Award. Medtronic PLC provided devices but no financial support.

## CONFLICT OF INTEREST

H.M.B.-S. serves on a clinical advisory board for Medtronic PLC.

All other authors have no conflict of interest to report.

## DATA AVAILABILITY

Data supporting the findings of this study will be made available on request when possible from the corresponding author.
